# Supplementary material for: WACSAW: An adaptive, statistical method to classify movement into sleep and wakefulness states
Source: PLoS One. 2025 Dec 11;20(12):e0333417. doi: 10.1371/journal.pone.0333417 (PMC12698012; doi:10.1371/journal.pone.0333417)
Supplement: S1 Appendix — (DOCX) [file pone.0333417.s001.docx]

## Hyperparameter Grid Search

The final WACSAW algorithm contains 4 hyperparameters to which we assigned values as we developed the WACSAW process. These assignments were based on quick trial and error, but they needed to be formally tuned to determine what values optimize WACSAW output. We used data from the same 6 training individuals with their varying sleep characteristics and tuned the model using accuracy to determine the hyperparameters that led to the best outcomes.

We tuned (1) the order of the Wasserstein distance (ρ in [Eq. 3](https://www.biorxiv.org/content/10.1101/2023.08.08.552395v2.full#disp-formula-3)); (2) the window size of data input; (3) the change point threshold for segmentation; and (4) the significance value of the Levene test.

For the order of the Wasserstein distance, we considered p ∈{1, 2}. For the window size, we considered windows of size 2, 5, 7 minutes. For the change point threshold, we considered the statistics mean, median, and α-trimmed mean with α = 0.25 for a 2-day period. For the Levene significant level, we checked significance levels between 0 and 1 ({ 10^−s^ : s ∈ [0, 81]) (note that the extremely small values were computed using arbitrary precision arithmetic). It may be more appropriate to treat this value, not as a significance level of a test, but as a parameter one could tune to accentuate the differences between sleep and wakeful segments, such as quiescent wakefulness.

We use parameter sweeps to test the hyperparameters. This revealed that WACSAW is not overly sensitive to variations in the hyperparameters within the search space, indicating that inadvertent deviations from optimal do not change the final output of WACSAW to any fundamental extent. Comparing the accuracy of WACSAW to the adjusted log classifications revealed that the accuracy stayed above 90% for all hyperparameter combinations on the training set and standard deviation ranged from 1 to below 11, computed from the 6 individuals in the development set. Moreover, the standard deviation decreases as accuracy increases. The top performing combination was order 1 with a 5 minute window, a mean change point threshold, and a Levene significance level of 1e^-25^ (Table S1). But the top ten combinations all had an accuracy above 97% but all combinations also had a standard deviation in accuracy of 1.5-2% between individuals, which indicates that the adaptive aspects of WACSAW reduced the variability in accuracy sometimes observed when a common algorithm is applied between individuals.

There are three additional parameters that were set by visual inspection, namely *k*=20 and upper bound of 0.1 for the transport energy histograms, as well as the ξ cutoff of 7000 for the characteristic functions.
