# Supplementary figures and images for: WACSAW: An adaptive, statistical method to classify movement into sleep and wakefulness states
Source: PLoS One. 2025 Dec 11;20(12):e0333417. doi: 10.1371/journal.pone.0333417 (PMC12698012; doi:10.1371/journal.pone.0333417)

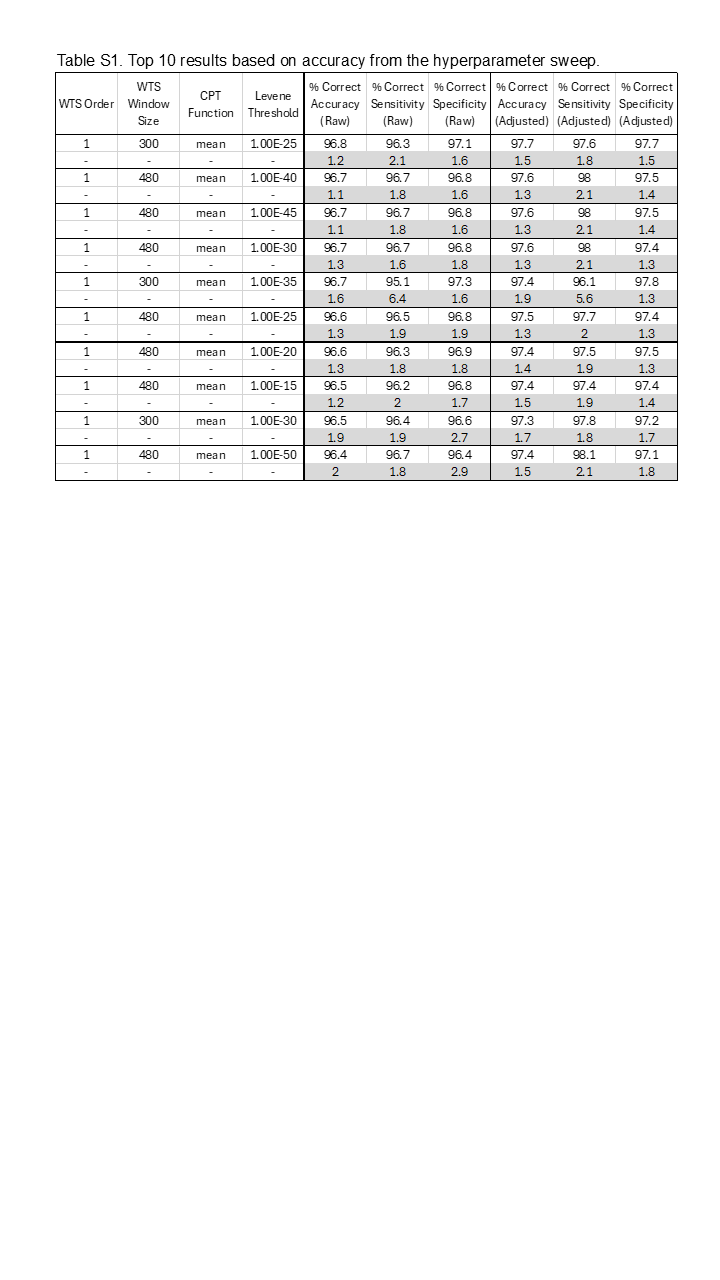

Supplement: S1 Table — (TIF) [file pone.0333417.s002.tif]

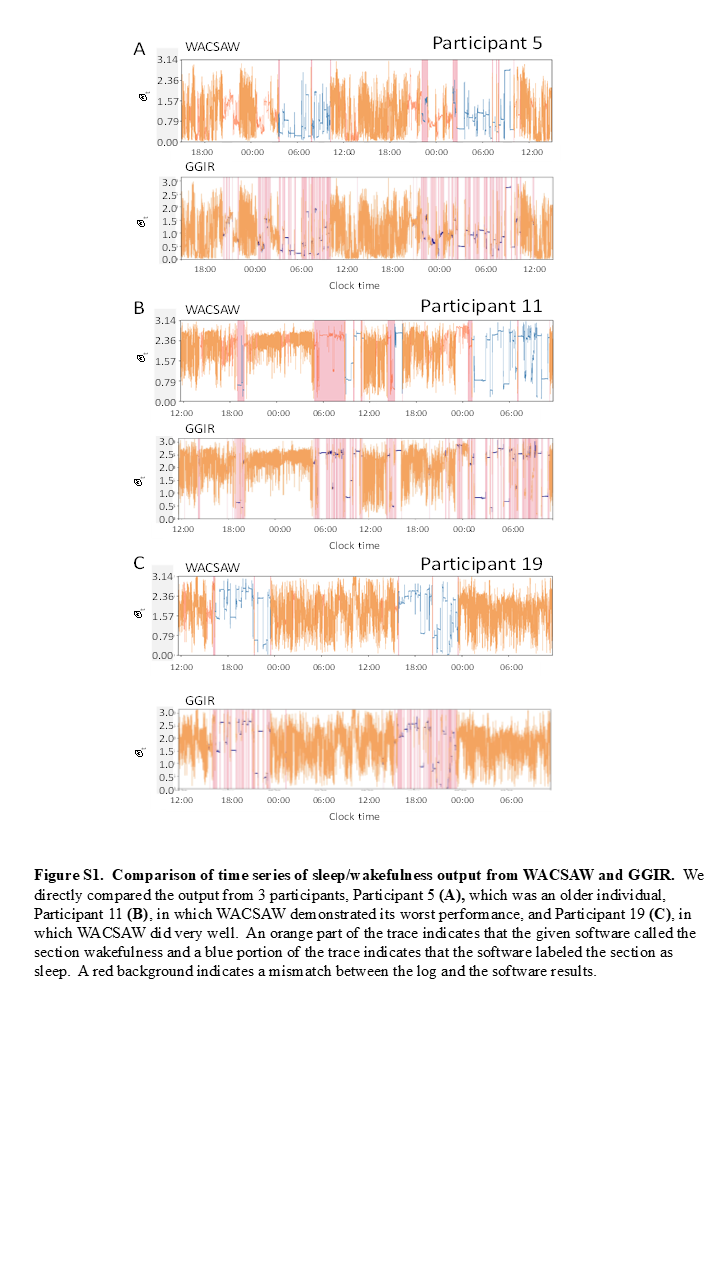

Supplement: S1 Fig — We directly compared the output from 3 participants, Participant 5 (A), which was an older individual, Participant 11 (B), in which WACSAW demonstrated its worst performance, and Participant 19 (C), in which WACSAW did very well. An orange part of the trace indicates that the given software called the section wakefulness and a blue portion of the trace indicates that the software labeled the section as sleep. A red background indicates a mismatch between the log and the software results. (TIF) [file pone.0333417.s003.tif]

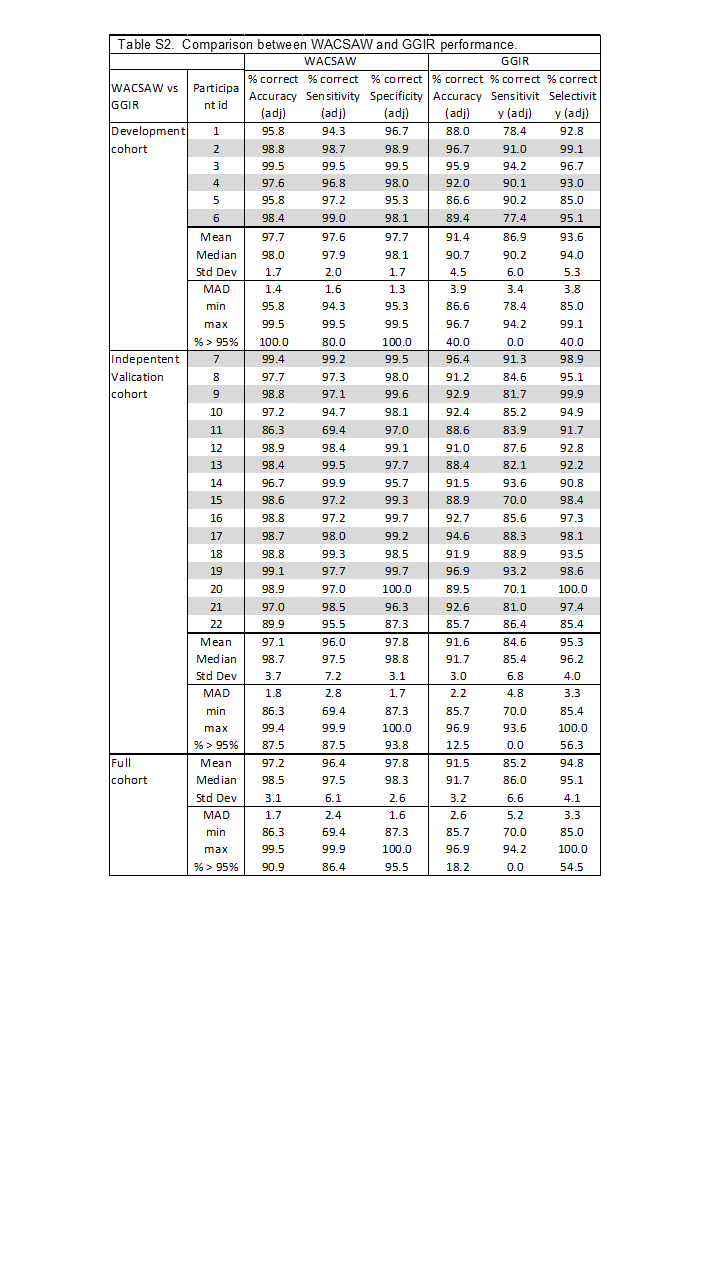

Supplement: S2 Table — (TIF) [file pone.0333417.s004.tif]

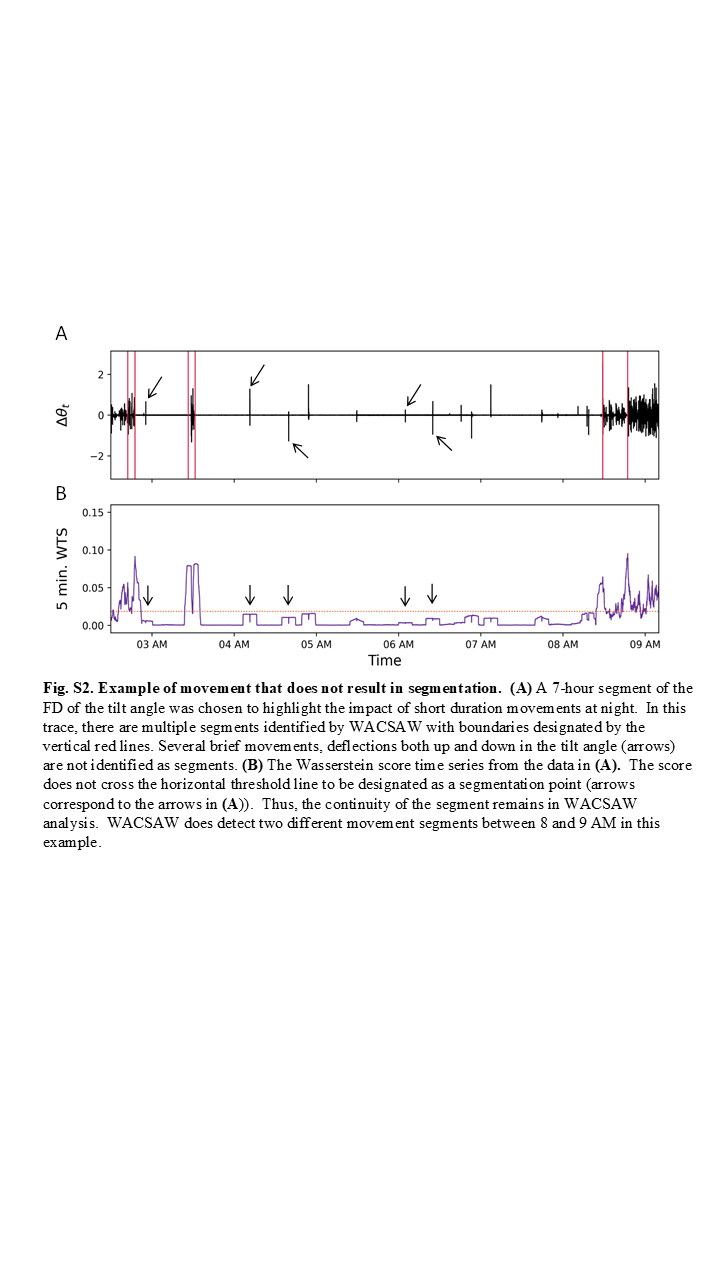

Supplement: S2 Fig — (A) A 7-hour segment of the FD of the tilt angle was chosen to highlight the impact of short duration movements at night. In this trace, there are multiple segments identified by WACSAW with boundaries designated by the vertical red lines. Several brief movements, deflections both up and down in the tilt angle (arrows) are not identified as segments. (B) The Wasserstein score time series from the data in (A). The score does not cross the horizontal threshold line to be designated as a segmentation point (arrows correspond to the arrows in (A)). Thus, the continuity of the segment remains in WACSAW analysis. WACSAW does detect two different movement segments between 8 and 9 AM in this example. (TIF) [file pone.0333417.s005.tif]
